# Are social protection and food security accelerators for adolescents to achieve the Global AIDS targets?

**DOI:** 10.1002/jia2.26369

**Published:** 2024-10-09

**Authors:** Lucie Cluver, Siyanai Zhou, Olanrewaju Edun, Allison Oman Lawi, Nontokozo Langwenya, David Chipanta, Gayle Sherman, Lorraine Sherr, Mona Ibrahim, Rachel Yates, Louise Gordon, Elona Toska

**Affiliations:** ^1^ Department of Social Policy and Intervention University of Oxford Oxford UK; ^2^ Department of Child and Adolescent Psychiatry University of Cape Town Cape Town South Africa; ^3^ Nuffield College University of Oxford Oxford UK; ^4^ Centre for Social Science Research University of Cape Town Cape Town South Africa; ^5^ School of Public Health and Family Medicine University of Cape Town Cape Town South Africa; ^6^ MRC Centre for Global Infectious Disease Analysis School of Public Health Imperial College London London UK; ^7^ Nutrition Division United Nations World Food Programme Rome Italy; ^8^ UNAIDS Windhoek Namibia; ^9^ Centre for HIV and STIs National Institute for Communicable Diseases Johannesburg South Africa; ^10^ Department of Molecular Medicine and Haematology Faculty of Health Sciences University of the Witwatersrand Johannesburg South Africa; ^11^ Department of Paediatrics and Child Health Faculty of Health Sciences University of the Witwatersrand Johannesburg South Africa; ^12^ Health Psychology Unit Institute of Global Health University College London London UK; ^13^ Department of Sociology University of Cape Town Cape Town South Africa

**Keywords:** adolescents, HIV prevention, prevention, stigma, social protection, treatment

## Abstract

**Introduction:**

Without effective, scalable interventions, we will fail to achieve the Global AIDS Targets of zero AIDS‐related deaths, zero HIV transmission and zero discrimination. This study examines associations of social protection and food security among adolescents living with HIV (ALHIV), with three Global AIDS Targets aligned outcomes: antiretroviral treatment (ART) adherence and viral suppression, HIV transmission risk behaviour and enacted stigma.

**Methods:**

We conducted three study visits over 2014−2018 with 1046 ALHIV in South Africa's Eastern Cape province. Standardized surveys provided information on receipt of government‐provided cash transfers and past‐week food security, alongside self‐reported ART adherence, sexual debut and condom use, and enacted HIV‐related stigma. Viral load (VL) data was obtained through data extraction from patient files and linkage with National Health Laboratory Service test results (2014−2020). We used a multivariable random‐effects regression model to estimate associations between receiving government cash transfers and food security and three outcomes: ART adherence and viral suppression, delayed sexual debut or consistent condom use and no enacted stigma. We tested moderation by sex and age and fitted disaggregated models for each outcome.

**Results:**

Among the 933 ALHIV completing all three study visits, 55% were female, and the mean age was 13.6 years at baseline. Household receipt of a government cash transfer was associated with improvements on all outcomes: ART adherence and viral suppression (aOR 2.03, 95% CI 1.29−3.19), delayed sexual debut or consistent condom use (aOR 1.62, 95% CI 1.16−2.27) and no enacted stigma (aOR 2.33, 95% CI 1.39−3.89). Food security was associated with improvements on all outcomes: ART adherence and viral suppression (aOR 1.73, 95% CI 1.30−2.30), delayed sexual debut or consistent condom use (aOR 1.30, 95% CI 1.03−1.64) and no enacted stigma (aOR 1.91, 95% CI 1.32−2.76). Receiving both cash transfers and food security increased the probability of ART adherence and VL suppression from 36% to 60%; delayed sexual debut or consistent condom use from 67% to 81%; and no enacted stigma from 84% to 96%.

**Conclusions:**

Government‐provided cash transfers and food security, individually and in combination, are associated with improved outcomes for ALHIV aligned with Global AIDS Targets. They may be important, and underutilized, accelerators for achieving these targets.

## 1 INTRODUCTION

The Global AIDS Strategy 2021−2026 aims to achieve the UNAIDS vision of zero AIDS‐related deaths, zero new HIV acquisitions and zero discrimination by 2030 [1]. But these goals remain elusive [2], particularly among the 1.7m adolescents living with HIV (ALHIV) globally, of whom 90% live in sub‐Saharan Africa [3]. This highly vulnerable group experiences low adherence to antiretroviral treatment (ART), inadequate viral suppression, low condom use, and ongoing stigma and discrimination [4, 5, 6]. Systematic reviews highlight a lack of evidence‐based services to improve these outcomes [7, 8, 9, 10].

Emerging evidence suggests progress, for example, recent randomized trials identify the effectiveness of peer‐led psychosocial support models [11] and family‐based economic empowerment interventions [12] in improving ART adherence and viral load (VL) suppression. However, systematic reviews find no interventions effectively reducing onward transmission of HIV or HIV‐related stigma and discrimination for adolescents in sub‐Saharan Africa [13, 14]. Additionally, no known research examines interventions that can simultaneously benefit all three global targets. With shrinking HIV budgets and social spending, exacerbated by COVID's economic sequelae [15], governments are seeking “development accelerators”: services that benefit multiple targets simultaneously [16].

South Africa has one of the widest‐reaching systems of social protection grants on the continent, including the government‐provided child support grant [17]. Social protection, especially cash transfers, and food security [18], may have the potential to be development accelerators for ALHIV. A recent analysis among people living with HIV in 42 countries [19] found that government cash transfer programmes were associated with improvements in antiretroviral coverage and reductions in AIDS‐related deaths and new HIV acquisitions. Evidence suggests that HIV‐related stigma is exacerbated by poverty [20, 21], and therefore, social protection may have a protective role [11]. Cross‐sectional studies suggest associations of social protection and food security with improved ART adherence for young people [22]. Among general populations of adolescents, social protection was associated with delayed sexual debut [23].

However, no known longitudinal research in sub‐Saharan Africa has examined the association of social protection and food security, independently and in combination, with ART adherence and viral suppression, HIV transmission risk behaviour and stigma experience among ALHIV. These HIV‐related outcomes can serve as indicators of progress towards the three zero Global AIDS targets among ALHIV. Global food price increases are intensifying food insecurity for vulnerable populations such as ALHIV, making the identification of effective interventions more vital than ever.

This study examines the associations of social protection and food security, independently and in combination, with multiple HIV outcomes related to Global AIDS targets, in a longitudinal cohort of ALHIV in South Africa.

## 2 METHODS

### 2.1 Study design and sample

We conducted a longitudinal cohort study of ALHIV in South Africa's Eastern Cape, a province with an HIV prevalence of 17% [24] and substantial healthcare system challenges [25]. Three study visits including baseline took place between 2014 and 2018. From mid‐2013 to early 2014, we identified all 52 facilities (eight hospital wards, five community health centres, 39 primary care clinics) providing HIV care to adolescents in Buffalo City District, Eastern Cape province [26]. At each facility, we reviewed patient files to identify all records for adolescents (10−19 years) who had initiated ART. All identified eligible ALHIV (*N* = 1176) were approached for recruitment into the study, at the health facilities or traced back to their home communities, to ensure that adolescents lost to follow‐up from the healthcare system were included at all study visits. Of the study‐eligible adolescents, 1046 were recruited (48 refused participation, 10 had cognitive delay too severe to give informed consent, 44 were untraceable and 28 no longer lived in the study area) and participated in the baseline visit in 2014−15. These adolescents were followed‐up for a second study visit in 2016−17, and a third study visit in 2017−18. The cohort had 94% retention at the second study visit follow‐up, and 91% retention at the third study visit follow‐up. Using both familyreport and National Health Laboratory Service data, 8.9% (94 out of 1046) of adolescents died over the full study period, with 13 deaths recorded in Wave 1, 33 in Wave 2 and 48 in Wave 3.

### 2.2 Study data and measures

At each study visit, data was collected from adolescents using tablet‐based standardized surveys. These assessed adolescents’ experiences in their homes, communities and healthcare settings, including information on ART adherence, sexual reproductive health and HIV stigma. Surveys were piloted with 25 adolescents, and designed to be non‐stigmatizing and engaging, through consultation with the study's adolescent advisory group. Adolescents completed the survey—in their homes, communities or at healthcare facilities—in their preferred language (English or isiXhosa), with the support of trained research assistants.

Primary outcomes were: the proportion of adolescents  who (i) reported past‐week ART adherence and were virally suppressed (VL<1000 copies/ml); (ii) reported delayed sexual debut or consistent condom use; and (iii) reported no enacted stigma experience in the past year.

ART adherence was collected using measures on missed doses with varying recall timeframes. Past‐week ART adherence was defined as a binary indicator of no missed doses in the past 7 days, and currently taking ART [27], based on items adapted from the Patient Medication Adherence Questionnaire developed in Botswana [28]. Patient files (paper‐based and electronic) were reviewed at each of the 52 healthcare facilities. Routine data, including plasma HIV VL, for each study participant, were extracted from paper‐based medical records at healthcare facilities, and supplemented with electronic laboratory test data from the National Health Laboratory Services data warehouse. Since VL measurements did not exactly align with self‐report interview dates, we used VL closest to the interview date (within 12 months) for each participant. *VL suppression* was defined as VL less than 1000 copies/ml.

Delayed sexual debut or consistent condom use was defined based on responses to items from the South African National HIV Survey [29], firstly assessing debut and then asking those who are sexually active “*In the last year, how often did you use condoms for the whole time that you were having sex?*”. Adolescents who responded “always” were coded as having consistent condom use, and all other responses were coded as not consistent. *Past year enacted stigma* was measured via the HIV‐stigma scale for ALHIV (ALHIV‐SS), developed in collaboration with ALHIV in South Africa, with strong psychometric properties [30]. *No past‐year enacted stigma* was defined as a negative response to all four items of the enacted stigma sub‐scale: “*My family mistreats me because of my HIV status”;* “*I have lost friends by telling them I have HIV”;* “*I have stopped spending time with some kids because of their reactions to my HIV status”; and* “*I've been teased because of my HIV status.”*

Measures on social protection provisions included receipt of government‐provided grants and food security. *Government cash grant* was defined based on whether an adolescent's household received any national government grant (child support, foster care, disability, pension or care dependency) [29]. *Food security* was defined based on positive responses to two items: (i) whether the household could afford meals in the past week and (ii) whether the adolescent had been able to have three meals per day, using national survey measures [31].

Nine covariates were measured: Adolescent *age* (divided into 10−14 and 15+ years age groups); *sex*; *residence* (urban/rural); *housing type* (informal/formal); *orphanhood* (paternal/maternal); and *household size* were measured using items adapted from the South African census*. Household poverty* was measured based on reported access to the eight highest socially perceived necessities for children and adolescents validated in a nationally representative South African survey [31]. *Mode of HIV acquisition* (recently vs. perinatally acquired HIV) was determined using clinic records, or following existing sub‐Saharan African paediatric cohorts which define perinatally acquired HIV as those initiating ART at ≤10 years old [32, 33], validated and updated with an algorithm that considered other variables (e.g. parental death) [34]. We measured *current intimate partner relationship status* using an item from the SA National HIV Survey [35].

### 2.3 Statistical analysis

We examined the frequencies of all measures at each study visit. We then examined associations between receiving government cash transfers and food security on the proportion of adolescents reporting past‐week ART adherence with suppressed VL (<1000 copies/ml), delayed sexual debut or consistent condom use and no experience of enacted stigma, using a multivariable random‐effects regression model suited to the repeated‐measures nature of the data. To aid the interpretation of relationships between the two predictors and each outcome, we estimated average adjusted probabilities of the association of each combination of main predictors on each outcome using *margins* commands in Stata. As a sensitivity check, we repeated the multivariate analysis above on all participants who provided data at any study visit (not only those who completed the survey at all study visits). We further fitted sex‐stratified and age‐group‐stratified models for each outcome, to ascertain if there were differences in associations with predictors. As a sensitivity check, we tested the moderation of associations by sex and age group (10−14 vs. 15+ years), respectively, for each outcome. A further sensitivity analysis was conducted to assess the impact of missingness in the combined self‐reported items and VL, using multiple imputation models. All parameters are estimated by maximum likelihood and with robust standard errors, clustered at the level of the individual using Stata v.16 software.

### 2.4 Ethics and informed consent

Ethical approvals were given by the University of Cape Town (UCT/CSSR/2013/4) and (UCT/CSSR/2019/01), University of Oxford (Oxford/CUREC2/12‐21), provincial Departments of Health and Education, National Health Laboratory Service Academic Affairs and Research Management System (2019/08/07), and ethical review boards of participating healthcare facilities. At all study visits, adolescent participants, and their caregivers when adolescents were <18 years old, provided voluntary, informed and written consent for participation, including interviews and access to adolescents’ clinical records. There were no financial incentives for study participation, but all participants received a small gift pack worth approximately $7.

## 3 RESULTS

Of the 1046 ALHIV who completed the survey at baseline, 94 died over the study period, and 933 completed the survey at all three study visits and were included in the main analyses. Those excluded from the analysis (*N* = 113, lost to follow‐up or died) were more likely to be older (mean age 14.5 vs. 13.5 years, *p*<0.001) and to be paternal orphans (40.7% vs. 28.9%, *p* = 0.010) than those retained (analysis sample).

Of the 933 ALHIV included in the main analyses, the mean age at baseline was 13.6 years, at the second visit 15.1 years, and at the third visit, 16.3 years (Table 1). Fifty‐five percent of the participants were female. Self‐reported past‐week ART adherence was low, ranging from 65% to 75% at each study visit, but in combination reaching only 37.1% sustained ART adherence over the study period [6]. Among those with a VL record at baseline (*n* = 746), the proportion with suppressed VL was 78%, at the second visit 80% (*n* = 656), and at the third visit, 74% (*n* = 578). Delayed sexual debut or past‐year consistent condom use was reported by 71% of adolescents at baseline, 76% at second visit and 78% at third visit. Experience of enacted stigma was 12% at baseline, 5% at second visit and 3% at third visit. Household receipt of government cash transfers was high (91% on average) across the three study visits, and past‐week food security was 77% of adolescents at baseline, 71% at second visit and 77% at third visit. Outcome measures were consistent across different study visits as shown by correlations in Table S1.

**Table 1:** Summary of participant characteristics (*n* = 933^a^ who completed three study visits)

	Study visit		
Variables	1 (2014−15) (*N* = 933)	2 (2016−17) (*N* = 933)	3 (2017−18) (*N* = 933)
Covariates	*n* (%)	*n* (%)	*n* (%)
Age (Mean/SD)	13.6 (2.9)	15.1 (2.9)	16.3 (2.9)
Female	514 (55)	514 (55)	514 (55)
Rural	249 (27)	230 (25)	223 (24)
Informal housing	172 (18)	134 (14)	131 (14)
Poverty	633 (68)	726 (78)	630 (68)
Recently acquired HIV	197 (21)	197 (21)	197 (21)
Paternal orphan	270 (29)	309 (33)	387 (41)
Maternal orphan	414 (44)	463 (50)	486 (52)
Household size (Mean/SD)	6.77 (2.90)	6.18 (3.82)	5.73 (2.96)
In a relationship	223 (24)	277 (30)	289 (31)
**Outcomes**			
ART adherence	615 (66)	605 (65)	700 (75)
Viral suppression^b^	582 (78)	527 (80)	427 (74)
Self‐reported adherence and virally suppressed	416 (56)	364 (55)	349 (60)
Delayed sexual debut or consistent condom use (past year)	667 (71)	708 (76)	725 (78)
No enacted stigma experience (past year)	819 (88)	855 (92)	868 (93)
**Main predictors**			
Any government cash grant	883 (95)	849 (91)	819 (88)
Food security	721 (77)	665 (71)	718 (77)

Abbreviation: SD, standard deviation.

^a^ *n* = 113 were excluded from the main analysis and comprised of (*n* = 12) who died between study visits 1 and 2, (*n* = 22) who died between study visits 2 and 3, (*n* = 55) who were lost‐to‐follow‐up (refusals, untraceable or avoidant) between study visits 1 and 2, and (*n* = 24) lost‐to‐follow‐up between study visits 2 and 3.

^b^ *n*1 = 746; *n*2 = 656; *n*3 = 578 (Number of adolescents with VL measurements at each study visit); Viral suppression was defined as VL<1000 copies/ml.

### 3.1 Associations of social protection factors with HIV outcomes

In multivariate analyses, both government cash transfers and food security were significantly associated with improvements on all three outcomes (Table 2). Controlling for age, sex, mode of HIV acquisition, rural residence, informal housing, poverty, paternal and maternal orphanhood, relationship status and household size, household receipt of government cash transfers was associated with higher odds of reporting past‐week ART adherence and suppressed VL (aOR: 2.03, 95% CI 1.29−3.19, *p* = 0.002), higher odds of delayed sexual debut or consistent condom use (aOR: 1.62, 95% CI 1.16−2.27, *p* = 0.004) and higher odds of no enacted stigma experience (aOR: 2.33, 95% CI 1.39−3.89, *p* = 0.001). Food security was also associated with higher odds of past‐week ART adherence and suppressed VL (aOR: 1.73, 95% CI 1.30−2.30, *p*<0.001), higher odds of delayed sexual debut or consistent condom use (aOR: 1.30, 95% CI 1.03−1.64, *p* = 0.028) and higher odds of no enacted stigma experience (aOR: 1.91, 95% CI 1.32−2.76, *p* = 0.001). Results for outcomes in which the sample is not restricted to those who completed the survey at all three study visits (*n* = 1046) showed similar results (Table S2).

**Table 2:** Multivariate random effects logistic regressions testing the impact of social protection on HIV outcomes (*n* = 933 adolescents, 2799 observations)

	Self‐reported adherence and virally suppressed (<1000 copies/ml)^a^		Delayed sexual debut and or consistent condom use (past year)		No enacted stigma experience (past year)	
Factors	aOR (95% CI)	*p*‐value	aOR (95% CI)	*p*‐value	aOR (95% CI)	*p*‐value
**Main predictors**						
Any gov't. grant	2.03 (1.29−3.19)	0.002	1.62 (1.16−2.27)	0.004	2.33 (1.39−3.89)	0.001
Food security	1.73 (1.30−2.30)	<0.001	1.30 (1.03−1.64)	0.028	1.91 (1.32−2.76)	0.001
**Covariates**						
Recently acquired HIV	0.54 (0.38−0.79)	0.001	0.35 (0.26−0.46)	<0.001	0.60 (0.39−0.93)	0.020
In a relationship	−	−	0.52 (0.41−0.67)	<0.001	−	−
Older adolescents (>15 years)	0.80 (0.61−1.05)	0.114	0.34 (0.26−0.45)	<0.001	1.03 (0.69−1.52)	0.904
Female	1.02 (0.78−1.34)	0.877	0.73 (0.59−0.90)	0.004	0.73 (0.52−1.01)	0.056
Rural residence	0.86 (0.64−1.15)	0.314	1.05 (0.82−1.35)	0.685	0.77 (0.54−1.10)	0.147
Informal housing	1.12 (0.80−1.56)	0.502	0.93 (0.70−1.23)	0.613	0.81 (0.52−1.28)	0.372
Poverty	0.96 (0.74−1.24)	0.741	1.01 (0.80−1.29)	0.878	1.03 (0.70−1.51)	0.884
Paternal orphan	0.87 (0.66−1.14)	0.319	1.01 (0.81−1.26)	0.929	0.80 (0.56−1.12)	0.191
Maternal orphan	1.07 (0.82−1.40)	0.631	1.02 (0.82−1.27)	0.850	0.77 (0.55−1.08)	0.126
Household size	1.01 (0.97−1.05)	0.539	0.99 (0.96−1.03)	0.843	1.01 (0.96−1.06)	0.657

^a^ Proportion of adolescents who reported ART adherence and were virally suppressed (<1000 copies/ml). For each of the models, we adjusted for study visit.

### 3.2 Adjusted probability of HIV‐related outcomes reflecting the impact of government cash transfers and food security

**Figure 1:**
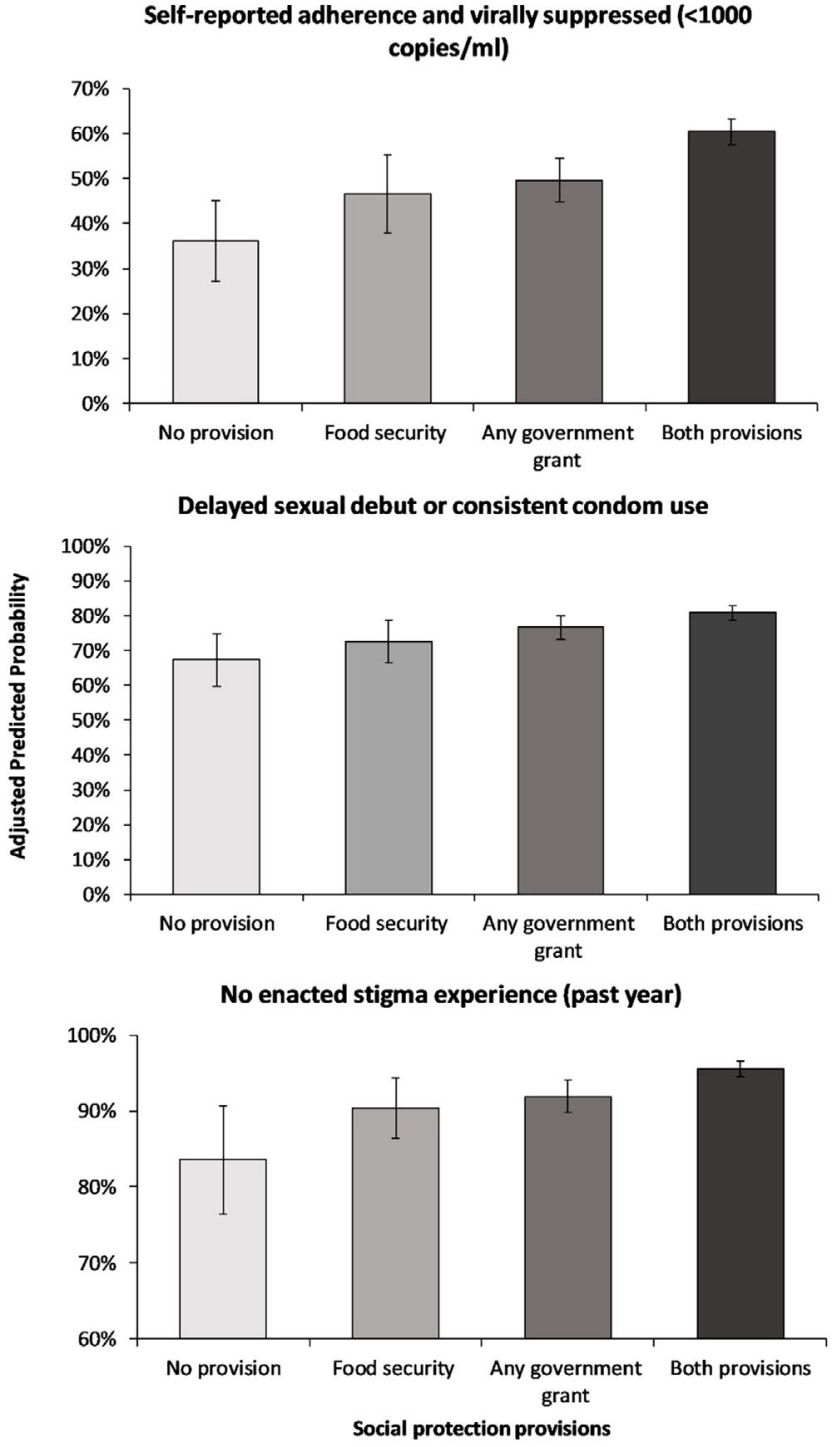
Adjusted predicted probability of combined self‐reported ART adherence and viral suppression (<1000 copies/ml), delayed sexual debut or consistent condom use, and enacted stigma experience.

The adjusted probability of a positive effect on all three outcomes was significantly higher for a scenario in which government cash transfers and food security were experienced together, as compared to a scenario with none or one of the two provisions (Figure 1). For combined past‐week ART adhernce and viral suppression, with neither government cash transfers nor food security, the adjusted probability was 36%. With both provisions, it was 60% (24 percentage points increase). For delayed sexual debut or consistent condom use, with neither government cash transfers nor food security, the adjusted probability was 67%; with both provisions, it was 81% (14 percentage points increase). For no enacted HIV stigma, with neither government cash transfers nor food security, the adjusted probability was 84%; with both provisions, it was 96% (12 percentage points increase).

### 3.3 Sex‐ and age‐stratified models for associations between social protection and HIV outcomes

**Figure 2:**
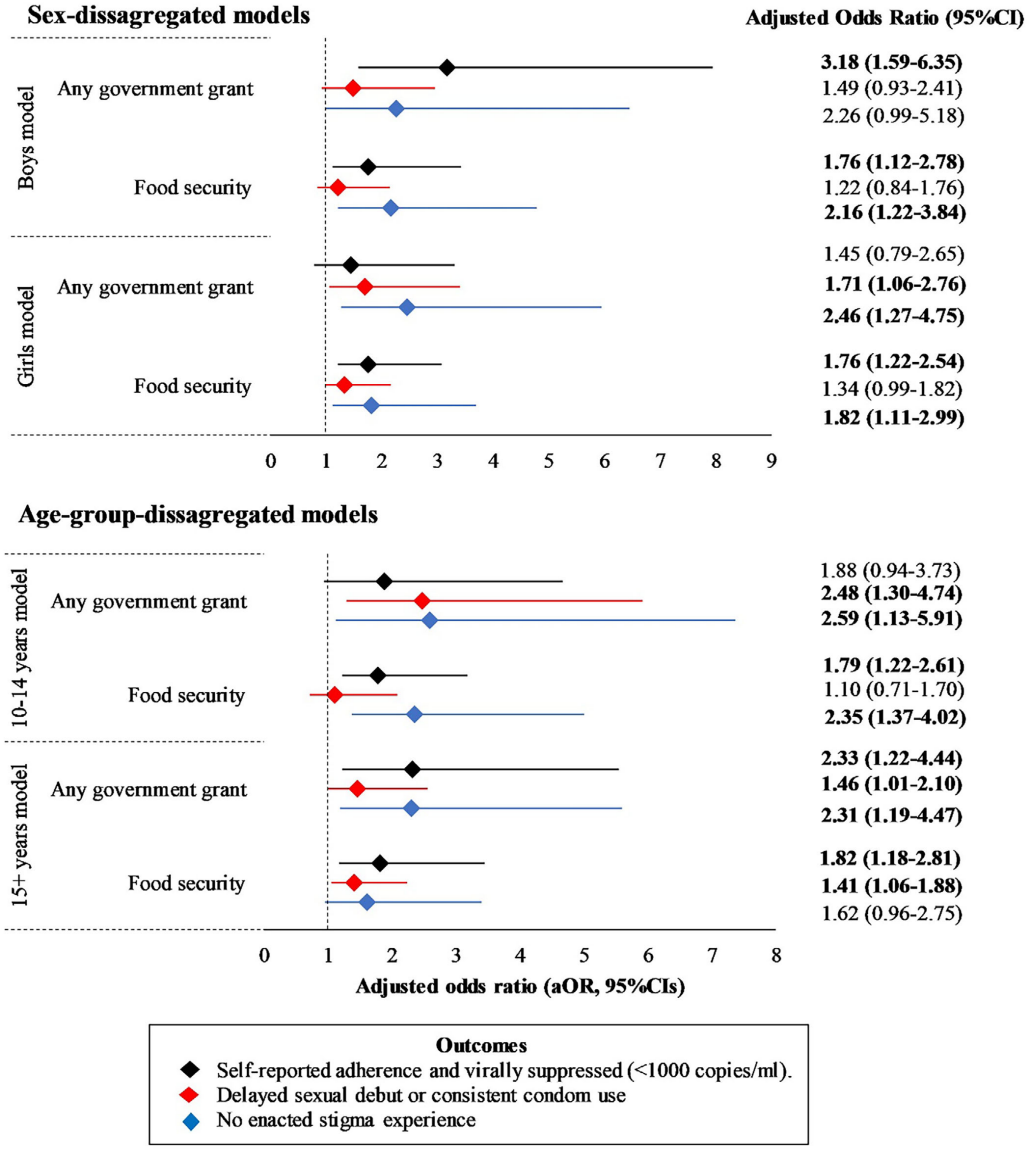
Sex‐ and age‐disaggregated models of the impact of social protection on HIV outcomes. *All models were adjusted for age, rural/urban residence, informal housing, poverty, orphanhood, household size, mode of HIV acquisition and current relationship status.

In sex‐stratified models, government cash transfers were significantly associated with higher odds of combined past‐week ART adherence and VL suppression for boys only (aOR: 3.18, 95% CI 1.59−6.35). Higher odds of delayed sexual debut or consistent condom use (aOR: 1.71, 95% CI 1.06−2.76) and no enacted stigma (aOR: 2.46, 95% CI 1.27−4.75) were observed for the effect of government cash transfer for girls only. Food security was significantly associated with higher odds of past‐week ART adherence and VL suppression for boys (aOR: 1.76, 95% CI 1.12−2.78) and girls (aOR: 1.76, 95% CI 1.22−2.54), and with higher odds of no enacted stigma for boys (aOR: 2.16, 95% CI 1.22−3.84) and girls (aOR: 1.82, 95% CI 1.11−2.99) (Figure 2). However we note that in statistical tests for moderation, sex did not show a significant interaction with government cash transfers or food security.

In models stratified by age, government cash transfers were significantly associated with higher odds of past‐week ART adherence and VL suppression for the 15+ years age group only (aOR: 2.33, 95% CI 1.22−4.44), and higher odds of no enacted stigma (aOR: 2.59, 95% CI 1.13−5.91 and aOR: 2.31, 95% CI 1.19−4.47) and delayed sexual debut or consistent condom use (aOR: 2.48, 95% CI 1.30−4.74 and aOR: 1.46, 95% CI 1.01−2.10) for both younger and older age‐groups, respectively. Food security was significantly associated with higher odds of combined past‐week ART adherence and VL suppression for younger (aOR: 1.79, 95% CI 1.22−2.61) and older adolescents (aOR: 1.82, 95% CI 1.18−2.81) and higher odds of no enacted stigma for younger adolescents (aOR: 2.35, 95% CI 1.37−4.02). Food security was significantly associated with higher odds of delayed sexual debut or consistent condom use for older adolescents (aOR: 1.41, 95% CI 1.06−1.88) (Figure 2).

### 3.4 Sensitivity analysis

Moderation models by sex and age group for each of the three outcomes did not show any significant interaction with government cash transfers or food security (Tables S3 and S4). A further sensitivity analysis assessing the impact of missingness in the VL measure on the relationship between government cash transfers, food security, and combined ART adherence and viral suppression showed similar effect sizes to the main analyses (Table S5).

## 4 DISCUSSION

We found that among a cohort of ALHIV in South Africa, government cash transfers and food security were independently associated with ART adherence and viral suppression, delayed sexual debut or consistent condom use and no enacted stigma. Combining government cash transfers and food security increased the probability of all three outcomes. Scaling‐up social protection in the form of (i) government cash transfers and (ii) interventions to promote food and nutritional security within adolescents’ households has the potential to improve HIV outcomes for ALHIV in South Africa.

Our findings are similar to results from Ghana which found a reduction in non‐adherence among 35 ALHIV receiving economic incentives. However, they found no change in viral suppression or perceived stigma [36]. Similarly, a cluster randomized trial in Uganda found higher self‐reported adherence among ALHIV receiving a family economic empowerment intervention [12]. Research on associations between government grants or food security and HIV transmission risk behaviour among ALHIV is sparse, but delayed sexual debut and reduced transactional sex have been found among general adolescent populations following economic empowerment in studies in Zambia [37] and among young sex workers in Zimbabwe [38].

Wider literature suggests several possible mechanisms whereby social protection and food security can influence HIV outcomes [39]. Studies show that households use government cash transfers to acquire food security as a priority [40]. There is evidence that social protection improves outcomes such as mental health [41], that may support ART adherence and prevention of onward transmission. By mitigating HIV‐related economic inequalities, social protection may reduce stigma related to poverty, and increase nutrition, cognitive development and access to education for adolescents living in HIV‐affected households [42]. Importantly, social protection may improve adolescents’ capacity to access HIV‐prevention resources, such as condoms [43], and also reduce the necessity for transactional sexual relationships to alleviate extreme poverty [44].

Findings suggest stronger impacts on ART adherence and VL suppression for adolescent boys and older adolescents, groups with particularly high rates of ART default [45], and on safe sexual behaviours for older adolescents (with sexual activity increasing by age) and adolescent girls, who experience re‐infection, transmission and high rates of adolescent pregnancy, with associated risks to their children [46].

Within South Africa, barriers to receipt of national grants include loss or lack of identity documents (e.g. related to parental death or delays in registration systems), lack of citizenship status, low literacy and knowledge of support systems, and living in areas with overburdened systems. Older adolescents age out of child support grant remit at 18 years. Research has shown that community organizations can effectively improve access to social protection for HIV‐affected families [47]. Despite national cash transfers, high rates of food insecurity remain across South Africa, with grants for children and pensioners often unable to support extended families [48].

Key lessons of this study are the importance of ensuring the inclusion of ALHIV into social protection programmes, and the added value of “cash plus” approaches combining social protection with other interventions [49]. Very often, programmes do not focus on adolescents, and instead, are targeted towards young children or pregnant and breastfeeding women. Adolescents have caloric requirements that far exceed the 2100 kcal used as a basic calculation for food baskets—often by 300−500 kcal per day—and thus, even within programmes, do not have their basic needs met. Enrolment of ALHIV in social protection schemes, including food‐based programming and school feeding, could make a crucial contribution in achieving Global AIDS targets. To achieve this, social protection programming may need either deliberate inclusion of ALHIV within the determination of vulnerable groups, allowance for age‐related needs within programme design or active identification and linkage to existing generic social protection programmes [43, 50]. HIV programming may need to play catalytic and linking roles, by supporting or initiating the inclusion of ALHIV in social protection programmes, while removing barriers to accessing these programmes.

We note several study limitations. First, the observational design requires caution in determining causality. However, longitudinal cohort data, including three study visits over 4 years, allowed advanced statistical modelling with increased internal validity to detect associations between potential protective factors and HIV outcomes. Experimental designs to further explore causality could take place at a local level, for example randomizing economic support for families when adolescents initiate ART, or at a national level, such as when Kenya rolled out its World Bank‐supported orphaned and vulnerable children cash transfer using a cluster randomized wait‐list approach [23]. Second, in our cohort, access to social protection and adequate food may have been pre‐existing for many adolescents and these as well as other unmeasured confounders may have contributed to our results. Research in high‐poverty contexts shows that social protection and food access need to be sustained and regular to improve health [19, 51]. Consequently, our assessment of current provision over a 4‐year period has value in understanding adolescent HIV outcomes, but future research could examine the impacts of sustained provision throughout pregnancy, childhood and adolescence. Third, self‐reported HIV outcomes may be subject to social desirability bias, although we combined self‐reported ART adherence with clinical measures of viral suppression. Fourth, this study is limited to a single country, therefore, generalizability across the region or to other resource‐limited settings is unknown. Lastly, our measure of food security focused on household capacity to afford meals, and future research should consider broader interpretations of food security, including adequate nutritional intake, and our measure of social protection focused on government‐provided support, and further research should examine the impacts of other sources of social protection, for example from non‐governmental organisations.

## 5 CONCLUSIONS

Our study assessed whether social protection and food security are associated with ART adherence and viral suppression, delayed sexual debut or consistent condom use and enacted stigma among ALHIV, proxies of the zero Global AIDS targets. Findings suggest that a poverty‐targeted government cash transfer, and access to enough food, are independently and over time associated with improvements in all three outcomes of increased ART adherence and viral suppression, increased safe sexual behaviours and reduced experience of enacted stigma. As ALHIV continue to face multiple challenges, we must prioritize their access to antiretroviral medication and equally prioritize assessing and addressing social protection and food security to give ALHIV the essential resources that facilitate their capacity to survive and thrive.

## COMPETING INTERESTS

The authors have no conflict of interest to report.

## AUTHORS’ CONTRIBUTIONS

LC conceptualized the study; SZ and LC conducted analyses and wrote the first draft; ET led the overall research study; ET, OE, GS and SZ led the conceptualization and analysis of the linked VL data; RY, NL and DC contributed an understanding of social protection and HIV; MI and LS contributed an understanding of stigma and treatment; AOL contributed an understanding of social protection and food security/nutrition; and LG supported writing and editing. All co‐authors read, reviewed and contributed to the writing of the final manuscript.

## FUNDING

This study was funded by the Swedish International Development Agency (SIDA)’s joint regional programme 2gether 4 SRHR through UNICEF's Eastern and Southern Africa Office; UK Research and Innovation Global Challenges Research Fund (UKRI GCRF) Accelerating Achievement for Africa's Adolescents (Accelerate) Hub [ES/S008101/1]; Oak Foundation (R46194/AA001, OFIL‐20‐057); Nuffield Foundation [CPF/41513]; Evidence for HIV Prevention in Southern Africa, a UKAID programme managed by Mott MacDonald; Janssen Pharmaceutica NV part of the Janssen Pharmaceutical Companies of Johnson & Johnson; and the International AIDS Society through the CIPHER grant (155‐Hod; 2018/625‐TOS); Claude Leon Foundation (08 559/C); the John Fell Fund (103/757 and 161/033); the University of Oxford's Economic and Social Research Council Impact Acceleration Account (IAA‐MT13‐003; 1602‐KEA‐189; K1311‐KEA‐004); the Leverhulme Trust (PLP‐2014‐095); Research England; the European Research Council (ERC) under the European Union's (EU) Seventh Framework Programme (FP7/2007‐2013)/ERC grant agreement 313421, the EU's Horizon 2020 research and innovation programme/ERC grant agreement 737476, 771468); the UK Medical Research Council (MRC) and the UK Department for International Development (DFID) under the MRC/DFID Concordat agreement, and by the Department of Health and Social Care through its National Institutes of Health Research (MR/R022372/1|), the Fogarty International Center, National Institute on Mental Health, National Institutes of Health under Award Number K43TW011434.

## DISCLAIMER

The content is solely the responsibility of the authors and does not represent the official views of the National Institutes of Health.

## Supporting information




**Table S1**: Correlations between outcomes measures across different study visits.
**Table S2**: Multivariate random effects logistic regressions testing the impact of social protection on HIV outcomes (without restricting the sample to those who were interviewed at all three study visits).
**Table S3**: Multivariate random‐effects logistic regressions testing the moderation effect of sex on the impact of social protection on adolescents' HIV‐related outcomes (n = 933 people, 2799 observations).
**Table S4**: Multivariate random‐effects logistic regressions testing the moderation effect of age (10–14 vs 15+ years age groups) on the impact of social protection on adolescents' HIV‐related outcomes (n = 933 people, 2799 observations).
**Table S5**: Multiple imputation results of the association between social protection, and self‐reported ART adherence and VL suppression.

## Data Availability

The data that support the findings of this study are available for no‐profit use from the corresponding author, Lucie Cluver, upon reasonable request.

## References

[1] UNAIDS . End inequalities. End AIDS. Global AIDS Strategy 2021–26. Geneva: UNAIDS; 2021.

[2] UNAIDS . In Danger: UNAIDS Global AIDS Update 2022. Geneva: UNAIDS; 2022.

[3] UNAIDS . Global AIDS Estimates. 2021.

[4] Pantelic M , Casale M , Cluver L , Toska E , Moshabela M . Multiple forms of discrimination and internalized stigma compromise retention in HIV care among adolescents: findings from a South African cohort. J Int AIDS Soc. 2020;23(5):e25488. 32438498 10.1002/jia2.25488 PMC7242009

[5] Toska E , Zhou S , Laurenzi CA , Haghighat R , Saal W , Gulaid L , et al. Predictors of secondary HIV transmission risk in a cohort of adolescents living with HIV in South Africa. AIDS. 2022;36(2):267–276. https://www.ncbi.nlm.nih.gov/pmc/articles/PMC8702447/ 34342294 10.1097/QAD.0000000000003044 PMC8702447

[6] Zhou S , Cluver L , Shenderovich Y , Toska E . Uncovering ART adherence inconsistencies: an assessment of sustained adherence among adolescents in South Africa. J Int AIDS Soc. 2021;24(10):e25832. 34708912 10.1002/jia2.25832 PMC8552454

[7] Casale M , Carlqvist A , Cluver L . Recent interventions to improve retention in HIV care and adherence to antiretroviral treatment among adolescents and youth: a systematic review. AIDS Patient Care STDs. 2019;33(6):237–252. 31166783 10.1089/apc.2018.0320 PMC6588099

[8] Laurenzi CA , Du Toit S , Ameyan W , Melendez‐Torres G , Kara T , Brand A , et al. Psychosocial interventions for improving engagement in care and health and behavioural outcomes for adolescents and young people living with HIV: a systematic review and meta‐analysis. J Int AIDS Soc. 2021;24(8):e25741. 34338417 10.1002/jia2.25741 PMC8327356

[9] Munyayi FK , van Wyk B , Mayman Y . Interventions to improve treatment outcomes among adolescents on antiretroviral therapy with unsuppressed viral loads: a systematic review. Int J Environ Res Public Health. 2022;19(7):3940. 35409621 10.3390/ijerph19073940 PMC8997420

[10] Reif LK , Abrams EJ , Arpadi S , Elul B , Mcnairy ML , Fitzgerald DW , et al. Interventions to improve antiretroviral therapy adherence among adolescents and youth in low‐ and middle‐income countries: a systematic review 2015–2019. AIDS Behav. 2020;24(10):2797–2810. 32152815 10.1007/s10461-020-02822-4 PMC7223708

[11] Mavhu W , Willis N , Mufuka J , Bernays S , Tshuma M , Mangenah C , et al. Effect of a differentiated service delivery model on virological failure in adolescents with HIV in Zimbabwe (Zvandiri): a cluster‐randomised controlled trial. Lancet Glob Health. 2020;8(2):e264–e275. 31924539 10.1016/S2214-109X(19)30526-1

[12] Ssewamala FM , Dvalishvili D , Mellins CA , Geng EH , Makumbi F , Neilands TB , et al. The long‐term effects of a family based economic empowerment intervention (Suubi+Adherence) on suppression of HIV viral loads among adolescents living with HIV in southern Uganda: findings from 5‐year cluster randomized trial. PLoS One. 2020;15(2):e0228370. 32040523 10.1371/journal.pone.0228370 PMC7010288

[13] Pantelic M , Steinert JI , Park J , Mellors S , Murau F . ‘Management of a spoiled identity’: systematic review of interventions to address self‐stigma among people living with and affected by HIV. BMJ Glob Health. 2019;4(2):e001285. 10.1136/bmjgh-2018-001285 PMC6441299 30997170

[14] Toska E , Pantelic M , Meinck F , Keck K , Haghighat R , Cluver L . Sex in the shadow of HIV: a systematic review of prevalence, risk factors, and interventions to reduce sexual risk‐taking among HIV‐positive adolescents and youth in sub‐Saharan Africa. PLoS One. 2017;12(6):e0178106. 28582428 10.1371/journal.pone.0178106 PMC5459342

[15] UNICEF Innocenti Office of Research . COVID‐19 and shrinking finance for social spending. Florence: UNICEF; 2022.

[16] UNDP . SDG accelerator and bottleneck assessment. New York: UNDP; 2017.

[17] ILO WFP , UNAIDS . HIV sensitive social protection in East and Southern Africa fast track countries. Geneva: ILO; 2021;67–70.

[18] Chipanta D , Pettifor A , Edwards J , Giovenco D , Topazian HM , Bray RM , et al. Access to social protection by people living with, at risk of, or affected by HIV in Eswatini, Malawi, Tanzania, and Zambia: results from population‐based HIV impact assessments. AIDS Behav. 2022. 10.1007/s10461-022-03645-1 PMC8938650 35316470

[19] Richterman A , Thirumurthy H . The effects of cash transfer programmes on HIV‐related outcomes in 42 countries from 1996 to 2019. Nat Hum Behav. 2022;6(10):1362–1371. 35851840 10.1038/s41562-022-01414-7

[20] Chipanta D , Stöckl H , Toska E , Chanda P , Mwanza J , Kaila K , et al. Facing the quality of life: physical illness, anxiety, and depression symptoms among people living with HIV in rural Zambia—a cross‐sectional study. AIDS Care. 2022:34(8):957–965. 34383600 10.1080/09540121.2021.1966693

[21] Philip AA , King J , Durham J . Lived experiences of persons with disabilities living with HIV in accessing HIV services in Africa: a qualitative systematic review. Disabil Rehabil. 2022. 10.1080/09638288.2022.2051079 35298321

[22] Cluver LD , Toska E , Orkin FM , Meinck F , Hodes R , Yakubovich AR , et al. Achieving equity in HIV‐treatment outcomes: can social protection improve adolescent ART‐adherence in South Africa? AIDS Care. 2016;28(**Suppl** 2):73–82. 27392002 10.1080/09540121.2016.1179008 PMC4991216

[23] Handa S , Halpern CT , Pettifor A , Thirumurthy H . The government of Kenya's cash transfer program reduces the risk of sexual debut among young people age 15–25. PLoS One. 2014;9(1):e85473. 24454875 10.1371/journal.pone.0085473 PMC3893206

[24] Johnson L , Dorrington R . Modelling the impact of HIV in South Africa's provinces: 2024 update. Centre for Infectious Disease Epidemiology and Research Working Paper. 2024.

[25] Massyn N , Day C , Dombo M , Barron P , English R , Padarath A , et al. District Health Barometer 2012/13. Durban: Health Systems Trust; 2013.

[26] Cluver L , Pantelic M , Toska E , Orkin M , Casale M , Bungane N , et al. STACKing the odds for adolescent survival: health service factors associated with full retention in care and adherence amongst adolescents living with HIV in South Africa. J Int AIDS Soc. 2018;21(9):e25176. 30240121 10.1002/jia2.25176 PMC6149366

[27] Paterson DL , Swindells S , Mohr J , Brester M , Vergis EN , Squier C , et al. Adherence to protease inhibitor therapy and outcomes in patients with HIV infection. Ann Intern Med. 2000;133(1):21–30. 10877736 10.7326/0003-4819-133-1-200007040-00004

[28] Duong M , Piroth L , Grappin M , Forte F , Peytavin G , Buisson M , et al. Evaluation of the patient medication adherence questionnaire as a tool for self‐reported adherence assessment in HIV‐infected patients on antiretroviral regimens. HIV Clin Trials. 2001;2(2):128–135. 11590521 10.1310/M3JR-G390-LXCM-F62G

[29] Department of Social Development , SASSA , UNICEF . The South African Child Support Grant Impact Assessment: evidence from a survey of children, adolescents and their households. South Africa: UNICEF; 2012.

[30] Pantelic M , Boyes M , Cluver L , Thabeng M . ‘They say HIV is a punishment from god or from ancestors’: cross‐cultural adaptation and psychometric assessment of an HIV stigma scale for South African adolescents living with HIV (ALHIV‐SS). Child Indicators Res. 2018;11(1):207–223. 10.1007/s12187-016-9428-5 PMC5816760 29497463

[31] Pillay U , Roberts B , Rule SP , editors. South African social attitudes: changing times, diverse voices. HSRC press; 2006.

[32] CIPHER Global Cohort Collaboration . Inequality in outcomes for adolescents living with perinatally acquired HIV in sub‐Saharan Africa: a Collaborative Initiative for Paediatric HIV Education and Research (CIPHER) Cohort Collaboration analysis. J Int AIDS Soc. 2018;21:e25044. 29485724 10.1002/jia2.25044 PMC5978669

[33] Sherr L , Cluver L , Toska E , He E . Differing psychological vulnerabilities among behaviourally and perinatally HIV infected adolescents in South Africa–implications for targeted health service provision. AIDS Care. 2018;30(sup2):92–101. 29848010 10.1080/09540121.2018.1476664

[34] He E , Tolmay J , Zhou S , Saal W , Toska E . Mode of HIV acquisition among adolescents living with HIV in resource‐limited settings: A data‐driven approach from South Africa. PLoS One. 2023;18(2):e0281298. 36827268 10.1371/journal.pone.0281298 PMC9955664

[35] Reproductive Health Research Unit (University of the Witwatersrand) and Lovelife . The National Survey of HIV and Sexual Behaviour among Young South Africans. 2005.

[36] Galárraga O , Enimil A , Bosomtwe D , Cao W , Barker DH . Group‐based economic incentives to improve adherence to antiretroviral therapy among youth living with HIV: safety and preliminary efficacy from a pilot trial. Vulnerable Child Youth Stud. 2020;15(3):257–268. 33281920 10.1080/17450128.2019.1709678 PMC7717062

[37] Austrian K , Soler‐Hampejsek E , Behrman JR , Digitale J , Jackson Hachonda N , Bweupe M , et al. The impact of the Adolescent Girls Empowerment Program (AGEP) on short and long term social, economic, education and fertility outcomes: a cluster randomized controlled trial in Zambia. BMC Public Health. 2020;20:1–5. 32183783 10.1186/s12889-020-08468-0 PMC7079524

[38] Chabata ST , Hensen B , Chiyaka T , Mushati P , Musemburi S , Dirawo J , et al. The impact of the DREAMS partnership on HIV incidence among young women who sell sex in two Zimbabwean cities: results of a non‐randomised study. BMJ Global Health. 2021;6(4):e003892. 10.1136/bmjgh-2020-003892 PMC8088246 33906844

[39] World Food Programme . World Food Programme Strategy for Support to Social Protection. World Food Programme; 2021.

[40] Tiwari S , Daidone S , Ruvalcaba MA , Prifti E , Handa S , Davis B , et al. Impact of cash transfer programs on food security and nutrition in sub‐Saharan Africa: a cross‐country analysis. Glob Food Sec. 2016;11:72–83. 31396473 10.1016/j.gfs.2016.07.009 PMC6687324

[41] Cluver LD , Sherr L , Toska E , Zhou S , Mellins CA , Omigbodun O , et al. From surviving to thriving: integrating mental health care into HIV, community, and family services for adolescents living with HIV. Lancet Child Adolesc Health. 2022;6(8):582–592. 35750063 10.1016/S2352-4642(22)00101-8

[42] Mebrahtu H , Skeen S , Rudgard WE , Toit SD , Haag K , Roberts KJ , et al. Can a combination of interventions accelerate outcomes to deliver on the Sustainable Development Goals for young children? Evidence from a longitudinal study in South Africa and Malawi. Child Care Health Dev. 2022;48(3):474–485. 34907593 10.1111/cch.12948

[43] Mebrahtu H , Skeen S , Rudgard WE , Du Toit S , Haag K , Roberts KJ , et al. Reducing adolescent risky behaviors in a high‐risk context: the effects of unconditional cash transfers in South Africa. Econ Dev Cult Change. 2017;65(4):619–652.

[44] Cluver L , Rudgard WE , Toska E , Orkin M , Ibrahim M , Langwenya N , et al. Food security reduces multiple HIV infection risks for high‐vulnerability adolescent mothers and non‐mothers in South Africa: a cross‐sectional study. African Rev Reprod Gynaecol. Endosc. 2022;25(8):e25928. 10.1002/jia2.25928 PMC9411725 36008916

[45] Hlophe LD , Tamuzi JL , Shumba CS , Nyasulu PS . Barriers and facilitators to anti‐retroviral therapy adherence among adolescents aged 10 to 19 years living with HIV in sub‐Saharan Africa: A mixed‐methods systematic review and meta‐analysis. PLoS One. 2023;18(5):e0276411. 37200399 10.1371/journal.pone.0276411 PMC10194875

[46] Toska E , Laurenzi CA , Roberts KJ , Cluver L , Sherr L . Adolescent mothers affected by HIV and their children: a scoping review of evidence and experiences from sub‐Saharan Africa. Global Public Health. 2020;15(11):1655‐1673. 32507031 10.1080/17441692.2020.1775867 PMC7578028

[47] Thurman TR , Kidman R , Taylor T . Bridging the gap: the impact of home visiting programs on social grant uptake among HIV‐affected households in South Africa. Child Youth Serv Rev. 2015;48:111–116.

[48] De Wet‐Billings N . Perpetuation of household food insecurity during COVID‐19 in South Africa. Journal of Health, Population and Nutrition. 2023;42(1):96. 37700382 10.1186/s41043-023-00441-y PMC10498595

[49] UNAIDS . Global AIDS Strategy 2021–2026 — End Inequalities. End AIDS. UNAIDS: 2021.

[50] Toska E , Gittings L , Hodes R , Cluver L , Govender K , Chademana KE , et al. Resourcing resilience: social protection for HIV prevention amongst children and adolescents in Eastern and Southern Africa. Afr J AIDS Res. 2016;15(2):123–40. 27399042 10.2989/16085906.2016.1194299 PMC5558245

[51] Food and Agriculture Organisation . Food Security. Rome: FAO; 2006.

