# Supplementary material for: Are social protection and food security accelerators for adolescents to achieve the Global AIDS targets?
Source: J Int AIDS Soc. 2024 Oct 9;27(10):e26369. doi: 10.1002/jia2.26369 (PMC11464211; doi:10.1002/jia2.26369)
Supplement: Supplementary file 1 — Table S1: Correlations between outcomes measures across different study visits. Table S2: Multivariate random effects logistic regressions testing the impact of social protection on HIV outcomes (without restricting the sample to those who were interviewed at all three study visits). Table S3: Multivariate random‐effects logistic regressions testing the moderation effect of sex on the impact of social protection on adolescents' HIV‐related outcomes (n = 933 people, 2799 observations). Table S4: Multivariate random‐effects logistic regressions testing the moderation effect of age (10–14 vs 15+ years age groups) on the impact of social protection on adolescents' HIV‐related outcomes (n = 933 people, 2799 observations). Table S5: Multiple imputation results of the association between social protection, and self‐reported ART adherence and VL suppression. [file JIA2-27-e26369-s001.docx]

**Appendices**

**Table S1: Correlations between outcomes measures across different study visits**

| **Outcome** | **T1** | **T2** | **T3** |
| --- | --- | --- | --- |
| Self-reported adherence and virally suppressedT1 | 1 |  |  |
| Self-reported adherence and virally suppressedT2 | 0.352 | 1 |  |
| Self-reported adherence and virally suppressedT3 | 0.324 | 0.413 | 1 |
| Delayed sexual debut/ consistent condom use (past year)T1 | 1 |  |  |
| Delayed sexual debut/ consistent condom use (past year)T2 | 0.352 | 1 |  |
| Delayed sexual debut/ consistent condom use (past year)T3 | 0.357 | 0.419 | 1 |
| No enacted stigma experience (past year)T1 | 1 |  |  |
| No enacted stigma experience (past year)T2 | 0.169 | 1 |  |
| No enacted stigma experience (past year)T3 | 0.188 | 0.149 | 1 |

T1: Study visit 1 (baseline); T2: Study visit 2; T3: Study visit 3.

**Table S2: Multivariate random effects logistic regressions testing the impact of social protection on HIV outcomes (without restricting the sample to those who were interviewed at all three study visits)**

|  | **Self-reported adherence and virally suppressed (<1000 copies/ml)*** | | **Delayed sexual debut/ consistent condom use (past year)** | | **No enacted stigma experience (past year)** | |
| --- | --- | --- | --- | --- | --- | --- |
| **Factors** | **aOR (95%CI)** | **p-value** | **aOR (95%CI)** | **p-value** | **aOR (95%CI)** | **p-value** |
| **Main predictors** |  |  |  |  |  |  |
| Any government grant | **2.03 (1.28 - 3.19)** | **0.002** | **2.39 (1.46 - 3.91)** | **<0.001** | **1.54 (1.11 - 2.14)** | **0.010** |
| Food security | **1.73 (1.30 - 2.30)** | **<0.001** | **2.08 (1.46 - 2.97)** | **<0.001** | **1.30 (1.04 - 1.63)** | **0.020** |
| **Covariates** |  |  |  |  |  |  |
| Recently acquired HIV | **0.54 (0.37 - 0.79)** | **0.001** | **0.63 (0.41 - 0.95)** | **0.029** | **0.35 (0.27 - 0.47)** | **<0.001** |
| In a relationship |  |  |  |  | **0.53 (0.42 - 0.67)** | **<0.001** |
| Older adolescents (>15 years) | 0.80 (0.60 - 1.05) | 0.114 | 1.03 (0.70 - 1.49) | 0.894 | **0.37 (0.29 - 0.47)** | **<0.001** |
| Female | 1.02 (0.78 - 1.33) | 0.877 | 0.79 (0.57 - 1.08) | 0.140 | **0.71 (0.58 - 0.88)** | **0.001** |
| Rural | 0.86 (0.64 - 1.15) | 0.314 | 0.84 (0.59 - 1.18) | 0.322 | 1.09 (0.85 - 1.38) | 0.503 |
| Informal housing | 1.12 (0.80 - 1.56) | 0.502 | 0.81 (0.52 - 1.24) | 0.325 | 0.90 (0.69 - 1.17) | 0.425 |
| Poverty | 0.96 (0.73 - 1.24) | 0.741 | 1.02 (0.70 - 1.48) | 0.922 | 0.98 (0.78 - 1.24) | 0.893 |
| Paternal orphan | 0.87 (0.66 - 1.14) | 0.319 | 0.81 (0.58 - 1.12) | 0.201 | 1.04 (0.84 - 1.29) | 0.689 |
| Maternal orphan | 1.07 (0.81 - 1.39) | 0.631 | 0.79 (0.57 - 1.08) | 0.150 | 0.99 (0.78 - 1.22) | 0.897 |
| Household size | 1.01 (0.97 - 1.05) | 0.539 | 0.99 (0.95 - 1.04) | 0.938 | 0.99 (0.96 - 1.03) | 0.757 |
| **Observations** | 1,972 |  | 2,887 |  | 2,937 |  |
| **Number of participants** | 842 |  | 1,034 |  | 1,034 |  |

* Proportion of adolescents who reported ART adherence and were virally suppressed (<1000 copies/ml). For each of the models, we adjusted for Study visit.

**Table S3: Multivariate random-effects logistic regressions testing the moderation effect of sex on the impact of social protection on adolescents’ HIV-related outcomes (n=933 people, 2799 observations).**

|  | **Self-reported adherence and virally suppressed (<1000 copies/ml)** | | **Delayed sexual debut/ consistent condom use (past year)** | | **No enacted stigma experience** **(past year)** | |
| --- | --- | --- | --- | --- | --- | --- |
| **Factors** | **aOR (95% CI)** | **p-value** | **aOR (95% CI)** | **p-value** | **aOR (95% CI)** | **p-value** |
| **Main predictors** |  |  |  |  |  |  |
| Any government grant | **3.24 (1.65-6.36)** | **0.001** | 1.44 (0.88-2.37) | 0.147 | 2.01 (0.87-4.63) | 0.100 |
| Food security | **1.83 (1.20-2.79)** | **0.005** | 1.17 (0.80-1.72) | 0.413 | **2.01 (1.14-3.52)** | **0.015** |
| **Interaction terms** |  |  |  |  |  |  |
| Any government grant # Female | 0.42 (0.17-1.04) | 0.060 | 1.25 (0.65-2.42) | 0.497 | 1.24 (0.45-3.42) | 0.672 |
| Food security # Female | 0.90 (0.52-1.56) | 0.711 | 1.19 (0.74-1.90) | 0.474 | 0.92 (0.46-1.84) | 0.818 |
| **Controls** |  |  |  |  |  |  |
| Older adolescents | 0.79 (0.60-1.05) | 0.099 | **0.33 (0.26-0.43)** | **<0.001** | 1.04 (0.70-1.53) | 0.854 |
| Female | 2.50 (0.92-6.80) | 0.073 | 0.51 (0.25-1.06) | 0.071 | 0.63 (0.21-1.89) | 0.414 |
| Rural residence | 0.86 (0.64-1.15) | 0.308 | 1.04 (0.83-1.32) | 0.719 | 0.77 (0.54-1.10) | 0.153 |
| Informal housing | 1.14 (0.81-1.59) | 0.452 | 0.91 (0.69-1.20) | 0.500 | 0.82 (0.53-1.29) | 0.400 |
| Poverty | 0.95 (0.73-1.23) | 0.673 | 1.00 (0.80-1.26) | 0.968 | 1.02 (0.70-1.50) | 0.911 |
| Paternal orphan | 0.86 (0.65-1.13) | 0.276 | 1.01 (0.82-1.26) | 0.906 | 0.79 (0.56-1.12) | 0.189 |
| Maternal orphan | 1.07 (0.82-1.41) | 0.603 | 1.04 (0.84-1.29) | 0.688 | 0.76 (0.55-1.06) | 0.108 |
| Household size | 1.01 (0.97-1.05) | 0.554 | 0.99 (0.96-1.03) | 0.740 | 1.01 (0.96-1.06) | 0.662 |
| Recently acquired HIV | **0.54 (0.37-0.79)** | **0.001** | **0.36 (0.28-0.47)** | **<0.001** | **0.60 (0.39-0.91)** | **0.018** |
| In a relationship | - | - | **0.51 (0.41-0.64)** | **<0.001** | - | - |

**Table S4: Multivariate random-effects logistic regressions testing the moderation effect of age (10-14 vs 15+ years age groups) on the impact of social protection on adolescents’ HIV-related outcomes (n=933 people, 2799 observations).**

|  | **Self-reported ART adherence and virally suppressed (<1000 copies/ml)** | | **Delayed sexual debut/ consistent condom use (past year)** | | **No enacted stigma experience** **(past year)** | |
| --- | --- | --- | --- | --- | --- | --- |
| **Factors** | **aOR (95% CI)** | **p-value** | **aOR (95% CI)** | **p-value** | **aOR (95% CI)** | **p-value** |
| **Main predictors** |  |  |  |  |  |  |
| Any government grant | 1.84 (0.88-3.82) | 0.104 | **2.56 (1.41-4.65)** | **0.002** | **2.79 (1.19-6.55)** | **0.018** |
| Food security | **1.77 (1.20-2.62)** | **0.004** | 1.00 (0.66-1.51) | 0.982 | **2.06 (1.23-3.46)** | **0.006** |
| **Interaction terms** |  |  |  |  |  |  |
| Any government grant # Younger adolescents (10-14 years) | 1.19 (0.45-3.12) | 0.728 | 0.56 (0.28-1.10) | 0.092 | 0.75 (0.27-2.10) | 0.589 |
| Food security # Younger adolescents (10-14 years) | 0.95 (0.56-1.60) | 0.846 | 1.47 (0.90-2.38) | 0.122 | 0.86 (0.44-1.70) | 0.667 |
| **Controls** |  |  |  |  |  |  |
| Older adolescents | 0.70 (0.25-1.98) | 0.502 | **0.43 (0.19-0.95)** | **0.036** | 1.48 (0.47-4.62) | 0.501 |
| Female | 1.02 (0.78-1.34) | 0.879 | **0.71 (0.58-0.88)** | **0.002** | 0.72 (0.52-1.01) | 0.055 |
| Rural residence | 0.86 (0.64-1.15) | 0.313 | 1.03 (0.81-1.32) | 0.785 | 0.77 (0.54-1.09) | 0.145 |
| Informal housing | 1.15 (0.82-1.60) | 0.419 | 0.91 (0.69-1.20) | 0.510 | 0.82 (0.52-1.29) | 0.387 |
| Poverty | 0.95 (0.74-1.24) | 0.726 | 0.99 (0.78-1.25) | 0.946 | 1.02 (0.69-1.49) | 0.935 |
| Paternal orphan | 0.86 (0.65-1.13) | 0.270 | 1.01 (0.82-1.26) | 0.896 | 0.79 (0.56-1.12) | 0.183 |
| Maternal orphan | 1.08 (0.82-1.41) | 0.584 | 1.04 (0.84-1.29) | 0.695 | 0.76 (0.54-1.06) | 0.106 |
| Household size | 1.01 (0.97-1.05) | 0.578 | 1.00 (0.96-1.03) | 0.857 | 1.01 (0.96-1.07) | 0.627 |
| Recently acquired HIV | **0.55 (0.38-0.80)** | **0.002** | **0.36 (0.27-0.47)** | **<0.001** | **0.59 (0.39-0.91)** | **0.017** |
| In a relationship | - | - | **0.51 (0.40-0.65)** | **<0.001** | - | - |

**Table S5: Multiple imputation results of the association between social protection, and self-reported ART adherence and VL suppression.**

|  | **Self-reported ART adherence and virally suppressed (<1000 copies/ml)^🗴^** | |
| --- | --- | --- |
| **Factors** | **aOR (95% CI)** | **p-value** |
| **Main predictors** |  |  |
| Any government grant | **1.88 (1.23-2.87)** | **0.003** |
| Food security | **1.54 (1.17-2.03)** | **0.002** |
| **Potential predictors** |  |  |
| Recently acquired HIV | **0.52 (0.37-0.72)** | **<0.001** |
| In a relationship |  |  |
| **Controls** |  |  |
| Older adolescents | 0.82 (0.63-1.05) | 0.120 |
| Female | 1.02 (0.79-1.32) | 0.871 |
| Rural residence | 0.82 (0.63-1.08) | 0.157 |
| Informal housing | 1.01 (0.74-1.39) | 0.941 |
| Poverty | 0.92 (0.73-1.17) | 0.506 |
| Paternal orphan | 0.91 (0.71-1.17) | 0.445 |
| Maternal orphan | 1.08 (0.84-1.38) | 0.565 |
| Household size | 1.00 (0.97-1.03) | 0.956 |

**^🗴^** Multiple imputation by chained equations was used to impute missing dichotomous elevated VL values. The imputation model included adolescent age, sex, mode of HIV acquisition, rural residence, informal housing, poverty, paternal and maternal orphanhood, and household size for each imputation model. The multivariable mixed-effects regression models were applied to 20 imputed data sets, and results were combined using Rubin's rules for each model. 95% CI – confidence interval.
